# Development and validation of the Chinese Rural Middle-aged and Elderly Health Literacy Scale

**DOI:** 10.1136/bmjph-2023-000797

**Published:** 2024-06-10

**Authors:** Zhengyu Wu, Wenjuan Gong, Yoshida Koki, Fei Wang, Yi-Chen Chiang, Maoming Jiang, Jiannan Ma, Jie Huang, Rui Li, Zhengkui Liu, Dewen Wang

**Affiliations:** 1 School of Public Affairs, Xiamen University, Xiamen, China; 2 School of Sociology and Anthropology, Xiamen University, Xiamen, China; 3 School of Medicine, Peking University, Beijing, Beijing, China; 4 School of Public Health, Xiamen University, Xiamen, China; 5 Xiamen University of Technology, Xiamen, China; 6 Guangdong Medical University, Zhanjiang, China; 7 Institute of Psychology Chinese Academy of Sciences, Beijing, China

**Keywords:** Public Health Practice, Public Health, Community Health

## Abstract

**Introduction:**

A notable deficiency lies in the absence of tailored instruments for comprehensively evaluating the health literacy of middle-aged and elderly residents in rural areas of China. Given the context of China’s ageing demographic and the increasing urban–rural disparities, it becomes imperative to formulate a dedicated assessment tool for appraising the health literacy of rural middle-aged and elderly populations.

**Methods:**

A systematic and rigorously structured development approach was used. First, the concept of health literacy was used to create a pool of items. Second, the Delphi method was used to revise and finalise the initial version of the Chinese Rural Middle-aged and Elderly Health Literacy Scale (CREHLS). Third, pretesting was used to assess the rationality of the item settings within the CREHLS. Finally, a large-sample survey was used to assess the reliability and validity of the finalisation CREHLS.

**Results:**

The CREHLS, which comprises 26 items distributed across four dimensions, is designed to evaluate the capacity to access, comprehend, assess and apply pertinent health information related to disease treatment, disease prevention, health promotion and environmental health. Multiple test results affirm the robust reliability and validity of the CREHLS, adhering to the established development standards.

**Conclusions:**

This study expects that the CREHLS will be validated in different rural regions of China and will be a reference for assessing the health literacy of rural middle-aged and elderly people in other countries, thereby increasing our attention to the health literacy of rural people.

**WHAT IS ALREADY KNOWN ON THIS TOPIC**

**WHAT THIS STUDY ADDS**

- In this context, the Chinese Rural Middle-aged and Elderly Health Literacy Scale (CREHLS) was designed. Our study found that the CREHLS demonstrates good reliability and validity, making it suitable for measuring the health literacy of middle-aged and elderly residents in rural China.

**HOW THIS STUDY MIGHT AFFECT RESEARCH, PRACTICE OR POLICY**

- This study develops a tool for assessing the health literacy of rural middle-aged and elderly individuals, thereby facilitating the measurement of their health literacy levels. Furthermore, it emphasises the significance of giving attention to the health literacy status of middle-aged and elderly populations residing in rural areas.

## Introduction

 Health literacy refers to the capacity of individuals to acquire, comprehend and apply fundamental health information and services, enabling them to make informed decisions concerning the maintenance and enhancement of their personal well-being.^1^ This proficiency strongly correlates with improved individual health outcomes, reduced chronic disease incidence, healthier lifestyle choices and increased utilisation of healthcare services.^2^,^4^ Hence, in the majority of nations, enhancing the health literacy of their populace stands as a pivotal policy goal due to its status as a fundamental, cost-effective and efficient strategy for augmenting the overall health of a country’s population. As scholarly interest in health literacy research continues to grow, there is an escalating demand for the creation of measurement tools in the field. Searches of literature platforms such as WOS, PubMed and Google Scholar show that between 1995 and 2004, research on health literacy measurement tools progressed relatively slowly. However, from 2005 onward, with heightened attention to the field of health literacy, research in the development of health literacy measurement tools began to experience rapid expansion. Numerous measurement tools were created, including the Demographic Assessment for Health Literacy,^5^ Health Literacy Skills Instrument,^6^ Health Literacy Questionnaire,^7^ European Health Literacy Survey Questionnaire (HLS-EU-Q),^8^ New Short-Form Health Literacy Instrument,^9^ eHealth Literacy Scale,^10^ Health Literacy Management Scale,^11^ among others. These tools have made diverse contributions to the progress of health literacy measurement to varying degrees. At the same time, on reviewing WOS and PubMed, we discovered that there remains a scarcity of health literacy scales specifically tailored for rural older adults in other countries. Nonetheless, a number of disease literacy scales exist for older adults. It is crucial to emphasise that disease literacy merely constitutes a subset of health literacy, thus limiting the comprehensive evaluation of the overall health literacy status of older adults.

In comparison to other nations, China embarked on its research concerning health literacy measurement tools relatively late, leaving substantial room for improvement. In 2008, the Chinese Ministry of Health convened a panel of medical and healthcare experts to compile the Health Literacy of Chinese Citizens: Basic Knowledge and Skills (Trial Edition). This document laid the foundation for the 66 essential elements of health literacy tailored to the Chinese populace. Simultaneously, the Chinese Health Education Center, an arm of the Ministry of Health, spearheaded the development of the Chinese Health Literacy Scale (CHLS), marking a significant milestone as China’s maiden officially developed health literacy measurement tool.^12^ The CHLS encompasses 6 dimensions, incorporating a total of 50 items. It is intended for monitoring the health literacy levels of Chinese residents between the ages of 15 and 69 years. According to the most recent findings derived from the Ministry of Health’s health literacy monitoring initiative,^13^ in 2022, the health literacy level of Chinese permanent residents aged 15–69 years attained 27.78%, with urban residents registering 31.94% and rural residents at 23.78%. These figures indicate that the health literacy level of Chinese residents remains comparatively low, with discernible disparities between urban and rural areas. In addition to the officially published health literacy measurement tools in China, Chinese scholars have also contributed to the development of health literacy measurement tools. These tools can be categorised into two primary types. One category involves the revision and enhancement of the CHLS to address its limitations and introduce new scales, such as the Revised CHLS.^14^ The other category entails the translation and adaptation of established measurement scales from other countries to create health literacy measurement tools suitable for Chinese residents or patients, as exemplified by the CHLS for Older People,^15^ Short-Form Health Literacy in Dentistry Scale^16^ and CHLS for Chronic Care.^17^ Nevertheless, the existing health literacy measurement tools in China are designed for assessing the health literacy of the general population or patients and do not encompass tools tailored for specific demographic groups, such as the middle-aged and elderly population residing in China rural areas.

In common with many developing countries, a substantial development gap exists between rural and urban regions in China. Nonetheless, due to historical factors, the rural–urban disparities in China are notably pronounced. From 1966 to 1976, China endured a decade of internal turmoil during which both the healthcare and scientific education systems in rural Chinese areas experienced varying degrees of disruption.^18^ Those residing in rural areas during this period had limited access to quality education and healthcare services, resulting in comparatively lower levels of education and health when compared with urban counterparts.^19^ Subsequent to the year 2000, China underwent rapid socioeconomic development with considerable government backing for healthcare and education. Nevertheless, the government’s strategy prioritised the development of urban areas. Consequently, residents in urban regions now have access to education and healthcare services akin to those in developed countries. Research indicates that rural areas in China continue to lag behind economically,^20^ with lower income levels and relatively inferior living conditions as opposed to urban areas. Educational resources in rural areas remain relatively scarce,^21^ resulting in lower educational attainment among rural residents. Furthermore, rural areas grapple with inadequate medical facilities and healthcare resources,^22^ leading to delayed access to healthcare services for rural residents. In light of this historical context, substantial disparities in terms of education levels, lifestyle habits and health status persist between urban and rural populations in China. Hence, using health literacy measurement tools intended for the entire population may present limitations, making it challenging to effectively address these differences with health literacy monitoring tools designed for the general population. According to data from the Seventh National Population Census,^23^ the ageing of China’s rural population is becoming increasingly severe. In the year 2020, the proportion of the population aged 60 years and over in rural areas had already surpassed 20%, and the share of the population aged 65 years and over had reached 17.72%, with the number of elderly people in rural areas totalling 121 million.^24^ The health of the rural population is a matter of utmost importance for the development of rural areas and the nation as a whole. Given the pronounced urban–rural development disparities in China, it holds great practical significance to focus on the health literacy of the elderly residing in rural areas. In light of the ongoing ageing of China’s population and the widening urban–rural divide, it is imperative to devise a specialised tool for gauging the health literacy levels of the middle-aged and elderly individuals in rural areas.

This article specifically offers a comprehensive overview of the structured and systematic methods employed for the item generation, pretesting and performance validation of the Chinese Rural Middle-aged and Elderly Health Literacy Scale (CREHLS). The primary goal is to create and assess a concept-based, multidimensional health literacy measurement tool tailored to the middle-aged and elderly population in rural China. Therefore, this article delves into the design and development process of the CREHLS, offering detailed descriptions of the methods used at each phase and presenting the results achieved in each stage. Finally, the article engages in a discussion regarding the applicability, quality and potential limitations of the CREHLS.

## Methodology

### Study setting and participants

We designed a systematic and rigorously structured approach to designing the CREHLS: (1) based on the concept of health literacy, we designed a 4x4 matrix with 16 cells, including dimensions such as disease treatment, disease prevention, health promotion and environmental health; (2) combining domestic and international research on health literacy, the item pool was established; (3) using methods such as the Delphi method to revise and finalise the initial version of the CREHLS; (4) underwent pretesting and a large sample survey to assess the reliability and validity of the CREHLS among rural middle-aged and elderly population. In the pretesting phase, the study conducted surveys among the rural middle-aged and elderly population in Fujian province (Quanzhou city) and Guangdong province (Shaoguan city) between January and March 2022. The survey used a convenient cluster sampling method. In the large sample survey stage, the study conducting surveys in Fujian province, including Quanzhou city, Zhangzhou city, Putian city and Fuzhou city, from July to September 2023. The survey employed a stratified cluster sampling method to select the survey areas.

### Study design

#### Applying a concept validation approach for design the CREHLS

Accurately defining the concept and framework of health literacy serves as a fundamental prerequisite for the development and design of the CREHLS.^25^ Sorensen provides the following definition for health literacy: ‘health literacy is linked to literacy and encompasses the motivation, knowledge and competencies necessary to access, understand, evaluate and apply health information to make informed decisions in daily life concerning disease treatment, disease prevention and health promotion, with the aim of preserving or enhancing the quality of life across the lifespan’.^26^ Drawing from this concept of health literacy, Sorensen introduced the conceptual framework for the HLS-EU-Q.^8^ The core of this framework can be visualised as a 12-cell matrix, featuring three dimensions along the vertical axis: disease treatment, disease prevention and health promotion. On the horizontal axis, four key aspects are integrated, covering access, understanding, evaluation and application in relation to health-related information. The concept and framework of health literacy introduced by Sorensen have garnered recognition among numerous scholars. Therefore, we adopt Sorensen’s concept of health literacy and proposes a conceptual framework model suitable for measuring health literacy among China’s rural middle-aged and elderly population. As depicted in table 1, this expansion results in a 4×4 matrix comprising 16 cells.

**Table 1:** Health literacy matrix

	Access	Understand	Appraise	Use
Disease treatment	The ability to obtain information about disease treatment or clinical issues	The ability to understand the disease treatment information obtained	The ability to appraise the scientific feasibility of the disease treatment information obtained	The ability to take action to better cope with disease treatment or recovery
Disease prevention	The ability to obtain information about disease prevention	The ability to understand the disease prevention information obtained	The ability to appraise the scientific feasibility of the disease prevention information obtained	The ability to take action to prevent diseases
Health promotion	The ability to obtain information related to improving health levels	The ability to understand the health-related information obtained	The ability to appraise the scientific feasibility of the health-related information obtained	The ability to take action to improve one's own health level
Environmental health	The ability to obtain information related to environmental health and hygiene	The ability to understand the environmental health information obtained	The ability to appraise the scientific feasibility of the environmental health information obtained	The ability to take action to improve one's living environment's hygiene and prevent environmental hazards to one's health

The disease treatment dimension focuses mainly on whether individuals can effectively obtain disease-related information when faced with clinical problems, whether they can understand the treatment plan and the importance of following doctors’ advice, and whether they can engage in self-management during the recovery process. Therefore, each item in this dimension aims to assess an individual’s ability to obtain and understand information related to disease management or clinical issues, and their ability to take action based on the information obtained to better cope with disease management or recovery. In contrast, the disease prevention dimension emphasises the prevention of disease through means such as changing unhealthy lifestyles, increasing awareness of personal hygiene and vaccination. Therefore, the items in this dimension aim to measure whether individuals have the ability to acquire and identify potential health risks and to take proactive preventive measures to reduce the risk of illness. Concurrently, the health promotion dimension focuses on how to encourage individuals to actively adopt healthier lifestyles or behaviours to maintain their level of health. This requires not only that individuals have the knowledge and skills to maintain their own health but also that they actively participate in various health promotion activities. Therefore, each item in this dimension aims to assess whether individuals can proactively engage in health-promoting behaviours, acquire more health knowledge and make healthier choices in their daily lives. In addition, the environmental health dimension emphasises that individuals should be aware of the impact of their environment on their health and take protective measures to reduce the impact of the environment on their health. This includes understanding the sources and hazards of environmental pollution, knowing how to improve indoor air quality and avoiding exposure to harmful environmental factors. Therefore, under this dimension, each item aims to assess whether individuals have the ability to acquire relevant information on environmental hygiene and whether they can, on the basis of the information acquired, take scientific action to improve the hygiene of their living environment and to prevent environmental hazards.

It is noteworthy that we incorporate an environmental health dimension, which is justified by three principal factors. First, individual health is intricately intertwined with the quality of their living environment. Factors such as air and water pollution can significantly elevate the risk of conditions such as cancer, cardiovascular diseases and respiratory illnesses, thereby markedly compromising residents’ overall health. Second, rural residents in China often possess limited understanding of the intricate relationship between the environment and health, lacking essential environmental awareness. Moreover, rural areas in China grapple with relatively weaker policies and control measures pertaining to environmental issues when compared with urban areas. Consequently, rural regions contend with a range of environmental challenges spanning water, soil and various other aspects.^27^

### Constructing the initial item pool for CREHLS

After clarifying the concept of health literacy and its core domains, we assembled a CREHLS development team consisting of three health experts, two instructors specialising in elderly health, three doctoral students and five master’s students. This collaborative team undertook the task of constructing the initial item pool for CREHLS. First, the CREHLS development team conducted interviews with a cohort of rural middle-aged and elderly individuals. Through a series of inquiries related to disease prevention, disease treatment, health promotion and other pertinent topics, the team garnered preliminary insights into the health literacy awareness among rural individuals. This valuable information served as a foundation for creating the initial item pool. For cost-efficiency and operational feasibility, the selection of rural middle-aged and elderly interviewees employed a convenience sampling method. Second, during the construction of the initial item pool for the CREHLS, the development team referenced existing health literacy, health status or health level measurement tools from both domestic and international sources.

### Delphi procedure for developing the preliminary version of CREHLS

The Delphi method is a technique employed in expert surveys, renowned for its extensive representation and reliability.^28^ It capitalises on the extensive knowledge and expertise of experts, often employing anonymous or face-to-face communication, allowing each expert to offer their assessments independently and candidly. To ensure the seamless progression of the Delphi process, our initial step involved reaching out to experts via email to ascertain their willingness and availability to participate and complete the Delphi procedure within the specified timeframe. Following the compilation of a list of participating experts for the Delphi activity, we disseminated the item pool to each expert, commencing the expert consultation process concerning the dimensions and items comprising the CREHLS. After several rounds of discussion, when consensus was reached among all the experts, the Delphi process was concluded and the preliminary version of the CREHLS was produced.

### Pretesting to assess the initial version of CREHLS

The development team collected small samples to validate and assess the initial version of CREHLS. This involved conducting a preliminary survey among rural middle-aged and elderly population in specific areas, namely Fujian province (Quanzhou city) and Guangdong province (Shaoguan city), between January and March 2022. The survey employed a convenient cluster sampling method. During this phase, the items of the initial version underwent a comprehensive analysis encompassing both quantitative and qualitative aspects, with guidance drawn from Robert’s scale development theory and operational procedures^29^: (1) frequency distribution analysis: to gauge the central tendency of item options, a frequency distribution analysis was employed. Items with excessively high selection frequencies (exceeding 70%) or low selection frequencies (where the cumulative frequencies for any two options fell below 10%) were evaluated for potential removal, taking practical considerations into account^30^; (2) dispersion trend analysis: dispersion trend analysis was employed to scrutinise the dispersion trends of item options. Items with lower dispersion trends indicate limited discriminative ability, implying that they may not effectively distinguish between individuals at different levels. Typically, if the coefficient of variation is less than 0.25 or the SD is less than 0.80 for an item, it signifies a reduced dispersion trend and warrants potential consideration for removal^31^; (3) Cronbach’s Alpha coefficient analysis: Cronbach’s α coefficient quantifies the proportion of the total variance in the test scores that results from the covariance among the items. It serves as an indicator of the internal consistency of the assessment content. During the computation of Cronbach’s α coefficient for each dimension of the scale, if the removal of a specific item enhances the α coefficient for its respective dimension, this implies that the presence of that item might diminish the internal consistency of the dimension, and therefore, its removal may be contemplated. In general, an α coefficient exceeding 0.7 is considered acceptable, whether it pertains to a specific dimension or the entire scale^32^; (4) corrected item-scale correlation analysis: corrected item-scale correlation evaluates the association between an item and the scale or its corresponding dimension, excluding the item under consideration. This correlation gauges the degree to which an item accurately reflects the scale or dimension. As a rule of thumb, when the corrected item-scale correlation falls below 0.40, it warrants consideration for removal^29^; (5) qualitative interview analysis: qualitative interview analysis was employed to collect feedback and recommendations from rural middle-aged and elderly participants. This feedback encompassed the comprehensibility, applicability, language expression habits and other dimensions of the scale items.

### Evaluation of the final version of CREHLS

Following the pretesting phase, the CREHLS development team conducted a comprehensive assessment of the items, considering the actual rural conditions and theoretical foundations. This assessment involved careful consideration of modifications, deletions and retention decisions, ultimately leading to the establishment of the final version of the CREHLS. In this version, all items are rated using a Likert scale, which allows respondents to express their actual states on a spectrum ranging from ‘very difficult’ to ‘difficult’, ‘moderate’, ‘easy’ and finally ‘very easy’.

The CREHLS development team conducted a large sample survey among the middle-aged and elderly population residing in rural areas, specifically targeting regions such as Zhangzhou, Putian and Fuzhou within Fujian province, from July to September 2023. The survey’s primary objective was to gather comprehensive data and evaluate the CREHLS’s performance in terms of reliability and validity. To ensure a representative sample and minimise potential sampling errors, the team employed a stratified cluster sampling method when selecting the survey areas.

### Data analysis

The data were coded before access to the software. The data were analysed using IBM SPSS V.26 software. Descriptive data were presented using tables. The level of significance was assessed at a 95% CI with a p<0.05.

## Results

### The CREHLS’s item pool

The CREHLS development team conducted interviews with 26 middle-aged and elderly individuals residing in rural areas, including 12 males and 14 females. Among these participants, 10 were within the age range of 45–59 years, while the remaining 16 were aged 60 years and above. After collecting the interview data, the team meticulously analysed the information, employing rigorous criteria to identify relevant items. Furthermore, the team drew inspiration from established health literacy scales, such as the HLS-EU-Q and CHLS, adapting and customising them to reflect the unique characteristics of rural China. For example, the CHLS includes an item pertaining to vaccines recommended for children post birth in accordance with the immunisation programme. Vaccination stands as the most effective and economical measure to prevent numerous diseases, such as influenza, thus safeguarding individuals from potential health risks. Given its crucial role, and inspired by the CHLS, we have modified the item on vaccines to cater to the needs of middle-aged and elderly population, who are also vulnerable to certain diseases and can greatly benefit from timely vaccination. These modified items were subsequently incorporated into the item pool. Using these methods, the CREHLS development team compiled a comprehensive repository consisting of 40 items, as detailed in online supplemental appendix 1.

### The initial version of CREHLS

A total of 20 experts were ultimately invited to participate, and their essential information is presented in online supplemental appendix 2. In the first Delphi round, a total of 20 expert consultation forms were distributed, and all 20 were returned, resulting in a response rate of 100%. However, during the data processing phase, the development team identified three experts whose forms had failed to provide any specific suggestions for modifications. This indicated a potential lack of in-depth evaluation on their part. Following careful deliberation and discussion, these three consultation forms were deemed invalid. Consequently, the effectiveness of expert input in the first round amounted to 85%. In the second Delphi round, 17 expert consultation forms were disseminated, excluding the three experts who were identified as not having diligently assessed the items in the first round. All 17 consultation forms received in the second round were deemed valid. In the Delphi process, the CREHLS development team adjusted or deleted items, guided by expert feedback and internal discussions among the development team members. A total of eight items were removed, primarily due to the majority of experts rating them as low in importance and appropriateness, along with providing detailed justifications for their exclusion. Additionally, 22 items underwent modifications due to issues such as unclear language, the amalgamation of core concepts or an excessive reliance on academic terminology. As a result, the initial version of the CREHLS was created with 34 items. Detailed information on the items can be found in online supplemental appendix 3.

### Pretesting results for the initial version of CREHLS

A total of 510 questionnaires were distributed to rural residents aged 45 years and above, and 491 questionnaires were subsequently returned, achieving a questionnaire return rate of 96.3%. Among these, 447 questionnaires were considered valid, resulting in an effective questionnaire rate of 91.0%. The demographic characteristics of the pretesting participants are described in table 2.

**Table 2:** The characteristics of the pretesting sample (n=447)

Characteristics	N (%)
Age (years)	
45–59	128 (28.6%)
More than 60	319 (66.9%)
Gender	
Male	187 (41.8%)
Female	260 (58.2%)
Education level	
Illiteracy	173 (38.7%)
Elementary school	154 (34.5%)
Middle school	71 (15.9%)
High school	29 (6.5%)
College or more	20 (4.5%)
Chronic disease	
Illness	299 (66.9%)
No illness	148 (33.1%)
Marital status	
Married	333 (74.5%)
Divorced or single	41 (9.2%)
Widowed	73 (16.3%)

Table 3 illustrates the frequency distribution of responses for each item in the initial version of the CREHLS. On analysis, we observed a pronounced skewness in the distribution of responses for options A8, B4, D7, D9 and D10. This skewness suggests that the distribution of responses is not representative or may bias the results, thereby warranting further consideration for their potential removal.

**Table 3:** Percentage of options for each item of the initial version CREHLS

Items	Percentage of options (%)				
	Very difficult	Difficult	Moderate	Easy	Very easy
A1	3.8	12.5	28.4	39.8	15.4
A2	3.6	14.3	29.1	39.4	13.6
A3	7.4	13.6	17.2	37.8	23.9
A4	2.7	14.5	27.7	42.3	12.8
A5	4.0	8.9	36.9	38.7	11.4
A6	7.2	16.1	36.5	29.3	11.0
A7	2.5	9.2	17.4	52.8	18.1
A8	2.0	3.4	23.0	44.5	27.1
B1	4.5	11.9	31.1	35.6	17.0
B2	2.0	9.4	26.8	48.1	13.6
B3	4.3	10.1	20.8	42.1	22.8
B4	1.6	5.8	11.6	47.2	33.8
B5	2.9	26.8	32.9	30.4	6.9
B6	3.1	9.2	38.5	42.3	6.9
B7	3.1	13.2	19.5	38.5	25.7
B8	4.5	7.6	26.0	41.2	20.8
C1	5.1	30.0	28.9	26.0	10.1
C2	6.7	17.7	30.0	36.2	9.4
C3	3.1	13.4	24.8	48.5	10.1
C4	5.4	13.0	25.7	42.1	13.9
C5	9.2	19.0	28.6	30.4	12.8
C6	3.1	6.9	27.1	47.9	15.0
C7	1.8	5.8	27.5	43.4	21.5
C8	5.8	32.4	39.4	17.4	4.9
D1	2.7	13.0	34.0	43.2	7.2
D2	2.7	8.5	25.3	53.2	10.3
D3	5.1	7.6	32.0	39.1	16.1
D4	2.0	14.5	24.6	40.0	18.8
D5	2.9	7.4	35.1	44.3	10.3
D6	1.8	6.9	13.9	43.8	33.6
D7	1.3	5.1	24.6	50.1	18.8
D8	4.5	6.7	30.0	20.1	38.7
D9	1.1	0.9	10.5	40.0	47.4
D10	1.1	2.5	11.6	48.3	36.5

Table 4 displays the mean scores, SD and coefficients of variation for each item in the initial version of the CREHLS. According to the criteria, items A8, B4, C7, D6, D7, D9 and D10 should be evaluated for potential removal.

**Table 4:** Degree of variation for each item of the initial version CREHLS

Items	Mean	SD	Variation coefficient	Items	Mean	SD	Variation coefficient
A1	3.51	1.02	0.29	C2	3.24	1.06	0.33
A2	3.45	1.01	0.29	C3	3.49	0.95	0.27
A3	3.57	1.20	0.34	C4	3.46	1.05	0.30
A4	3.48	0.98	0.28	C5	3.19	1.16	0.36
A5	3.45	0.95	0.28	C6	3.65	0.93	0.25
A6	3.21	1.07	0.33	C7	3.77	0.91	0.24
A7	3.75	0.94	0.25	C8	2.83	0.95	0.34
A8	3.91	0.90	0.23	D1	3.39	0.90	0.26
B1	3.49	1.05	0.30	D2	3.60	0.88	0.25
B2	3.62	0.91	0.25	D3	3.53	1.02	0.29
B3	3.69	1.62	0.29	D4	3.59	1.02	0.28
B4	4.06	0.91	0.22	D5	3.52	0.88	0.25
B5	3.12	0.98	0.31	D6	4.00	0.96	0.24
B6	3.41	0.87	0.25	D7	3.80	0.85	0.22
B7	3.70	1.08	0.29	D8	3.82	1.15	0.30
B8	3.66	1.03	0.29	D9	4.32	0.79	0.18
C1	3.06	1.08	0.35	D10	4.17	0.81	0.19

Table 5 provides a breakdown of the Cronbach’s alpha coefficients for each dimension in the initial version of the CREHLS. The alpha coefficients for the dimensions of disease treatment, disease prevention, health promotion and environmental health are 0.86, 0.80, 0.77 and 0.83, respectively. An analysis of the Cronbach’s alpha coefficients revealed that the removal of specific items had a relatively minimal positive effect on the alpha coefficient of their respective dimension. Consequently, the deletion of relevant items is not currently under consideration.

**Table 5:** Cronbach’s α of the initial version CREHLS

Items	α value after deleting an item	Items	α value after deleting an item
A1	0.838	B1	0.760
A2	0.834	B2	0.763
A3	0.838	B3	0.767
A4	0.843	B4	0.765
A5	0.836	B5	0.825
A6	0.870	B6	0.762
A7	0.853	B7	0.782
A8	0.859	B8	0.762
D1	0.817	C1	0.743
D2	0.812	C2	0.745
D3	0.824	C3	0.750
D4	0.813	C4	0.758
D5	0.805	C5	0.740
D6	0.814	C6	0.758
D7	0.813	C7	0.757
D8	0.818	C8	0.743
D9	0.809	–	–
D10	0.812	–	–

Table 6 displays the corrected item-scale correlations for each item in the initial version of the CREHLS. Considering the criterion, item B4 is currently under consideration for potential removal due to its relatively low correlation.

**Table 6:** Corrected item-scale correlation of the initial version CREHLS

Items	Corrected item-scale correlation	Items	Corrected item-scale correlation
A1	0.69	C2	0.65
A2	0.72	C3	0.61
A3	0.79	C4	0.59
A4	0.74	C5	0.68
A5	0.79	C6	0.56
A6	0.56	C7	0.57
A7	0.66	C8	0.65
A8	0.61	D1	0.61
B1	0.74	D2	0.66
B2	0.70	D3	0.61
B3	0.67	D4	0.68
B4	0.36	D5	0.72
B5	0.69	D6	0.59
B6	0.73	D7	0.64
B7	0.64	D8	0.70
B8	0.65	D9	0.68
C1	0.66	D10	0.65

The CREHLS development team actively interviewed with middle-aged and elderly individuals to solicit their feedback and opinions on the items. The results revealed concerns and valuable feedback from these individuals, particularly regarding items B4, B8, C1, D2, D4, D7, D9 and D10. Specifically, feedback indicated that some items were perceived as excessively simplistic while others were considered overly challenging. Consequently, the development team carefully consider modifying or removing these items in response to the feedback received.

### Evaluation results of the final version of CREHLS

After thoroughly synthesising the outcomes of analyses conducted during the pretesting phase, the CREHLS development team reached a unanimous decision to exclude a total of eight items: A8, B4, C2, C6, D4, D7, D9 and D10. Consequently, the final formal questionnaire was established, encompassing 4 dimensions and a total of 26 items, as detailed in online supplemental appendix 4.

The development team carried out a comprehensive performance assessment of the final version of CREHLS through a large sample survey. A total of 3081 questionnaires were distributed. Of these, 2606 valid questionnaires were collected, giving a questionnaire validity rate of 84.58%. The characteristics of the sample are shown in table 7.

**Table 7:** The characteristics of the large sample (n=2606)

Characteristics	N (%)
Age (years)	
45–59	1071 (41.6%)
More than 60	1536 (58.4%)
Gender	
Male	1335 (51.2%)
Female	1271 (48.8%)
Education level	
Illiteracy	802 (30.8%)
Elementary school	896 (34.4%)
Middle school	645 (24.8%)
High school	225 (8.6%)
College or more	38 (1.5%)
Chronic disease	
Illness	959 (36.8%)
No illness	1647 (63.2%)
Marital status	
Married	2350 (90.0%)
Divorced or single	34 (1.5%)
Widowed	222 (8.5%)

The reliability analysis presented in table 8 reveal that the Cronbach’s α for the final version CREHLS is 0.94. The Cronbach’s α for each dimension range from 0.78 to 0.84, indicating favourable internal consistency reliability. Additionally, the split-half reliability coefficient for the CREHLS is 0.91, with coefficients ranging from 0.76 to 0.84 for each dimension, further supporting the internal consistency of the CREHLS.

**Table 8:** Reliability of the final version CREHLS

Dimensions	Cronbach’s α	Split-half reliability
Disease treatment	0.82	0.80
Disease prevention	0.78	0.76
Health promotion	0.83	0.84
Environmental health	0.84	0.82

The structural validation of the final version of the CREHLS was carried out using confirmatory factor analysis. The results, presented in table 9, show that the model fit indices for the whole and each dimension meet the established standards, indicating a good structural validity of the CREHLS.

**Table 9:** Validity analysis of the final version CREHLS

Indicator	Integral	Disease treatment	Disease prevention	Health promotion	Environmental health
X^2^	2743.167	91.156	62.937	53.686	33.372
df	267.000	10.000	10.000	7.000	6.000
CFI	0.927	0.986	0.988	0.992	0.995
GFI	0.918	0.990	0.993	0.993	0.996
AGFI	0.892	0.972	0.981	0.979	0.985
TLI	0.911	0.971	0.974	0.982	0.988
RMSEA	0.058	0.056	0.045	0.051	0.041
SRMR	0.044	0.024	0.019	0.017	0.015

AGFIadjusted goodness of fit indexCFIcomparative fit indexGFIgoodness of fit indexRMSEAroot mean square error of approximationSRMRstandardized root mean square residualTLITucker Lewis index

Many studies have shown that individuals with higher levels of health literacy tend to have better health outcomes and a higher quality of life.^2^,^4^ When people are able to understand health information, navigate the healthcare system effectively and engage in preventive behaviours, they are more likely to make informed decisions about their health. This can lead to better management of chronic conditions, reduced healthcare costs and contributed to a higher quality of life. Therefore, the level of health literacy plays a significant role in shaping an individual’s health-related quality of life. The Short Form Health Survey (SF-12) is a widely used tool for assessing health-related quality of life. It evaluates various facets of health, encompassing bodily pain, general health, vitality, emotional role, physical functioning, mental health, physical health and overall health across eight dimensions. Higher scores on the SF-12 signify better health-related quality of life. Given the unique characteristics of rural China, adopting existing health literacy scales from other countries or populations as a calibration tool may not accurately reflect the actual situation in rural areas. On the other hand, health literacy is closely related to health-related quality of life, making it a potentially suitable option for this study. Therefore, adhering to the principles of criterion-related validity, the SF-12 was selected as the criterion measure for the CREHLS. The analysis of criterion validity demonstrates a substantial correlation between the CREHLS and SF-12, yielding a correlation coefficient of 0.35 (p<0.001). In summary, the criterion validity of the CREHLS satisfies the required standards.

## Discussion

The development process of the CREHLS follows a standardised procedure, using a variety of quantitative and qualitative research methods for item selection. The results indicate that the CREHLS meets the design standards for reliability and validity. The study reveals that the Cronbach’s α coefficient of the developed CREHLS is 0.94, with a split-half reliability of 0.91. The Cronbach’s α coefficients for each dimension range from 0.78 to 0.84, and the split-half reliabilities for each dimension range from 0.76 to 0.84, indicating good reliability. Additionally, the structural validity indices of the CREHLS meet the following criteria^33^: Tucker Lewis index (TLI)>0.900, comparative fit index (CFI)>0.900, adjusted goodness of fit index (AGFI)>0.900, standardized root mean square residual (SRMR)<0.050 and root mean square error of approximation (RMSEA)<0.050. Furthermore, the construct validity analysis demonstrates a moderate positive correlation (β=0.35, p<0.001) between CREHLS and SF-12, meeting the validation criteria of >0.3,^34^ further confirming the good validity of the CREHLS. The development of the CREHLS is firmly grounded in the field of public health, it considers the unique circumstances prevailing in rural China, with a focus on assessing the health literacy of middle-aged and elderly rural Chinese residents across four core domains: disease treatment, disease prevention, health promotion and environmental health. The CREHLS not only facilitates a more precise assessment of the health literacy among rural middle-aged and elderly rural individuals but also serves to heighten awareness of crucial health issues within vulnerable rural communities.

## Conclusion

This article offers a comprehensive overview of the design and development process of the CREHLS. The CREHLS has been conceived to serve as a reminder for researchers, prompting them to be mindful of the measurement biases that can arise from the unique characteristics of rural areas when assessing population health literacy. On the flip side, it has been designed to furnish a more precise measurement and monitoring instrument to enhance public health and population health in rural regions. Our aspiration is that this endeavour will stimulate greater attention and research dedicated to the health literacy of middle-aged and elderly individuals in rural areas.

### Limitations

Our study is subject to several limitations. First, we executed the pretesting solely in rural areas of three regions, whereas an ideal scenario would encompass a broader, stratified sampling approach across multiple regions. However, due to budgetary constraints, this was unfeasible for us. Second, during the pretesting stage, the sample exhibited a relatively small proportion within the age range of 45–59 years, while the demographic above the age of 60 years was comparatively larger. This distribution was influenced by the varying degrees of ageing within the surveyed area. Such disparities are likely to impact the representativeness of the sample during the pretesting.

## supplementary material

10.1136/bmjph-2023-000797 online supplemental file 1

## Data Availability

Data are available on reasonable request.
