# Supplementary material for: Development and validation of the Chinese Rural Middle-aged and Elderly Health Literacy Scale
Source: BMJ Public Health. 2024 Jun 10;2(1):e000797. doi: 10.1136/bmjph-2023-000797 (PMC11816413; doi:10.1136/bmjph-2023-000797)
Supplement: online supplemental file 1 [file bmjph-2-1-s001.pdf]

Appendix 1 The item pool

Disease treatment	1.Obtain the ability to find information about the treatment or rehabilitation of one's own illness.<br>2.Obtain the ability to find the correct response when faced with a sudden illness.<br>3.Understand the instructions accompanying one's own medications.<br>4.Understand the treatment or rehabilitation plan for one's own illness.<br>5.Judge the scientific validity of the disease treatment information obtained.<br>6.Judge the scientific validity of disease treatment plans proposed by others.<br>7.Can differentiate between psychological issues and mental illnesses.<br>8.Seek professional help when feeling unwell.<br>9.Adhere to medical prescriptions or use medication as per the instructions.<br>10.Avoid using antibiotics, anti-inflammatory drugs, and similar medications without proper guidance.
Disease prevention	11.Obtain disease prevention knowledge and information needed.<br>12.Find methods to prevent choking or falling accidents.<br>13.Understand the purpose and benefits of vaccination.<br>14.Understand the information provided on food packaging such as expiration dates.<br>15.Understand the health hazards of mold, spoilage, and rot in foods like grains, oils, rice, flour, peanuts, and soybeans.<br>16.Recognize that common psychological issues and mental illnesses can be prevented.<br>17.Judge the correctness of disease prevention knowledge and information obtained.<br>18.Take appropriate measures to prevent diseases.<br>19.Properly store medications according to storage requirements and avoid using expired or spoiled medications.
Health promotion	20.Obtain knowledge and information related to healthy lifestyles.<br>21.Obtain information related to medical policies for oneself or family members.<br>22.Obtain information related to elderly care policies for oneself or family members.<br>23.Understand the significance and benefits of regular health check-ups.<br>24.Understand the distinction between health supplements and medications.<br>25.Assess which health services provided by rural doctors or family doctors are beneficial for oneself or family members.<br>26.Judge which behaviors or habits in daily life can positively impact one's own or family members' health.<br>27.Adopt a healthy lifestyle to promote one's own or family members' well-being.<br>28.Seek help from professionals to promote one's own or family members' health.<br>29.Actively learn some psychological knowledge to enhance one's ability to cope with stress.
Environmental health	30.Find environmental health information related to dust, odors, sewage, noise, and other environmental issues when encountered.<br>31.Actively learn about policies and regulations related to environmental hygiene, such as garbage classification and straw burning.<br>32.Understand the health hazards posed by dust, odors, sewage, noise, and other environmental problems.<br>33.Understand the health hazards of dead poultry and pesticides.

34.Understand the benefits and purposes of separating garbage into recyclables, hazardous waste, and other waste categories.
35.Assess whether there is environmental pollution in one's living environment.
36.Comply with regulations prohibiting smoking in public places.
37.Safely and correctly use toxic substances like pesticides, insecticides, herbicides, and disinfectants.
38.Refrain from spitting in public places in daily life.
39.Maintain civilized and hygienic toilet habits and avoid defecating or urinating in public areas.
40.Manage human and animal waste hygienically in daily life.

Appendix 2 The characteristics of experts

Characteristics	First round	Second round
Age		
30 to 45	7(35.0%)	7(41.2%)
More than 45	13(65.0%)	10(58.8%)
Gender		
Male	8(40.0%)	5(29.4%)
Female	12(60.0%)	12(70.6%)
Professional title		
Middle title	3(15.0%)	3(17.6%)
Vice-senior title	6 (30.0%)	5(29.5%)
Senior Title	11(55.0%)	9(52.9%)
Profile		
Health specialist	5(25.0%)	4(23.4%)
General practitioner	2(10.0%)	2(11.8%)
Public health specialist	3(15.0%)	3(17.6%)
Psychologist	1(5.0%)	1(5.9%)
Sociologists	1(5.0%)	1(5.9%)
Senior rural health officer	4(20.0%)	2(11.8%)
Elderly care specialist	2(10.0%)	2(11.8%)
Rural doctors	2(10.0%)	2(11.8%)

Notes:(1) In China, the Middle title generally refers to Assistant Professor; the Vice-senior title, commonly known as Associate Professor; and the Senior title corresponds to Professor. (2) In China, a health specialist refers to a professional who is engaged in closely monitoring, analysing, and evaluating the health status and diseases of both populations and individuals. They are also involved in promoting health maintenance and health enhancement initiatives. While their role somewhat overlaps with that of dietitians in some countries, health specialists in China have a broader scope of responsibilities. (3) While China aims to ultimately develop rural doctors into general practitioners, this objective remains unrealized. Presently, rural doctors in China lack the requisite qualifications and training to function as general practitioners; their roles are akin to that of pharmacists.

Appendix 3 The initial version of CREHLS

Disease treatment	A1. Can access knowledge about disease treatment or rehabilitation.<br>A2. Can find the correct response when faced with sudden illnesses.<br>A3. Can understand the usage, dosage, and contraindications on medication labels.<br>A4. Can understand the disease treatment or rehabilitation plans provided by doctors.<br>A5. Can judge the scientific validity of disease treatment or rehabilitation knowledge obtained.<br>A6. Can distinguish between psychological issues and mental illnesses.<br>A7. Can proactively seek medical treatment at accredited medical institutions when feeling unwell.<br>A8. Can comply with medical advice or use medications correctly according to the medication labels.
Disease prevention	B1.Can access methods for disease prevention.<br>B2. Can access methods to prevent choking, falling, bedsores, burns, and other issues.<br>B3. Can understand the purpose and benefits of vaccination.<br>B4. Can understand the health hazards of consuming moldy and spoiled grains, oils, rice, and noodles.<br>B5. Can understand that common psychological issues and mental illnesses are preventable.<br>B6. Can judge the scientific validity of disease prevention methods or knowledge obtained.<br>B7. Can pay attention to food packaging labels, including expiration dates, when consuming food.<br>B8. Can properly store medicines in accordance with humidity, sunlight, or refrigeration requirements and avoid using expired or spoiled medications.
Health promotion	C1. Can understand family doctor policies, long-term care insurance, and other policies related to personal health.<br>C2. Can seek advice from village doctors or healthcare workers to improve personal or family health.<br>C3. Can acquire health-related knowledge from sources such as mobile phones and television.<br>C4. Can judge the scientific validity of health-related knowledge acquired.<br>C5. Can understand the difference between health foods and medicines.<br>C6. Can consume meals with a balanced diet, reduced salt and oil intake, and regular eating habits.<br>C7. Can undergo regular health check-ups at medical institutions.<br>C8. Can proactively learn some psychological knowledge to enhance resilience.
Environmental health	D1. Can find effective protective measures to protect personal health when living in a polluted environment.<br>D2. Can proactively understand policies related to garbage classification, straw burning, and environmental hygiene.<br>D3. Can understand the health hazards of water pollution, air pollution, soil contamination, and other factors.

	D4. Can understand the health hazards of diseased poultry, mosquitoes, flies, rats, cockroaches, and other factors.
	D5. Can understand the role and benefits of garbage classification in living environment and human health.
	D6. Can judge whether daily living environment is contaminated with environmental pollution such as air, water, noise, etc.
	D7. Can properly store and use toxic substances such as pesticides and disinfectants.
	D8. Can refrain from spitting in public places in daily life.
	D9. Can use hygienic toilets and avoid defecating in inappropriate places in daily life.
	D10. Can manage human and animal feces in a clean and hygienic manner in daily life.

Appendix 4 The final version of CREHLS

Disease treatment	1. Can find information about disease treatment or rehabilitation.<br>2. Can find the correct response when faced with sudden illnesses.<br>3. Can understand the usage, dosage, and contraindications on medication labels.<br>4. Can understand the treatment or rehabilitation plans provided by doctors.<br>5. Can determine the scientific validity of disease treatment or rehabilitation information obtained.<br>6. Can distinguish between psychological issues and mental illnesses.<br>7. Can proactively seek treatment at accredited medical institutions when feeling unwell.
Disease prevention	8. Can find methods for disease prevention.<br>9. Can find methods to prevent common risks such as choking, falling, bedsores, and burns.<br>10. Can understand the purpose and benefits of vaccination.<br>11. Can understand that common mental illnesses are preventable.<br>12. Can determine the scientific validity of disease prevention information obtained.<br>13. Can correctly store medicines as required and avoid using expired or spoiled medications.<br>14. Can check the expiration dates and other relevant information on food packaging when consuming.
Health promotion	15. Can proactively search for policies related to family doctors, long-term care, and other health-related matters.<br>16. Can proactively seek health-related knowledge.<br>17. Can understand the difference between health foods and medicines.<br>18. Can determine the scientific validity of health-related knowledge obtained.<br>19. Can regularly undergo health check-ups at medical institutions.<br>20. Can proactively learn about psychological health to enhance resilience.
Environmental health	21. Can find information about environmental hygiene policies such as garbage classification and straw burning.<br>22. Can understand the health hazards of water pollution, air pollution, soil pollution, etc.<br>23. Can understand the role and benefits of garbage classification in living environment and health.<br>24. Can determine whether there is environmental pollution in the living environment.<br>25. Can take effective measures to protect health when environmental pollution exists in daily life.<br>26. Can refrain from spitting or urinating in public places in daily life.
